# Salivary secretory leukocyte protease inhibitor levels in patients with stage 3 grade C periodontitis: a comparative cross-sectional study

**DOI:** 10.1038/s41598-022-24295-2

**Published:** 2022-12-08

**Authors:** Beral Afacan, Veli Özgen Öztürk, Gülnur Emingil, Timur Köse, Konstantinos Mitsakakis, Nagihan Bostanci

**Affiliations:** 1 Department of Periodontology, Faculty of Dentistry, Aydın Adnan Menderes University, Aydın, Turkey; 2 Department of Periodontology, Faculty of Dentistry, İzmir Ege University, İzmir, Turkey; 3 Department of Biostatistics and Medical Informatics, Faculty of Medicine, İzmir Ege University, İzmir, Turkey; 4 Hahn-Schickard, Freiburg, Germany; 5 Laboratory for MEMS Applications, IMTEK-Department of Microsystems Engineering, University of Freiburg, Freiburg, Germany; 6 Section of Oral Health and Periodontology, Division of Oral Diseases, Department of Dental Medicine, Karolinska Institutet, Alfred Nobels Allè 8, Huddinge, 14104 Stockholm, Sweden

**Keywords:** Periodontitis, Diagnostic markers

## Abstract

Secretory leukocyte protease inhibitor (SLPI) is an anti-protease that protects mucosal tissue integrity owing to its anti-microbial and immunomodulatory properties. This study aimed to investigate SLPI levels in periodontal diseases, and analyze the potential correlation with clinical periodontal parameters. Whole saliva samples were obtained from healthy (n = 24), gingivitis (n = 24) and patients with stage 3 grade C periodontitis (n = 24). SLPI was measured by ELISA and normalized by total protein. Receiver operating characteristics (ROC) curve was used for estimating the area under the curve (AUC). The normalized SLPI levels were significantly reduced in periodontitis compared with gingivitis (4.84-fold) or health (1.83-fold) and negatively correlated with periodontal parameters. The ROC curves showed a good predictor value of the SLPI for differentiation of periodontitis versus health or gingivitis (AUC ≥ 0.80). This study demonstrates that the levels of SLPI are high in periodontal health, further elevated in gingivitis, but eventually decreased in severe periodontitis beyond the former two states. This observation may have broader implications in the context of inflammatory diseases affecting the oral mucosa, as it shows that the bacterial burden is disturbing the homeostatic balances of anti-microbial and anti-protease factors in the oral cavity.

## Introduction

Periodontitis is a biofilm-driven chronic inflammatory disease that is characterized by irreversible destruction of tooth supporting periodontal tissues. While biofilm accumulation on tooth surfaces is necessary to initiate inflammation around the gingiva, periodontal tissue damage is a result of reciprocally reinforced interactions between the dysbiotic microbiome and the exacerbated host inflammatory response^1^.

The tissue-destructive host inflammatory response is mediated by the expression of diverse molecules including proinflammatory cytokines and proteolytic enzymes^2^. It is now evident that these molecules hold a key role in the emerging concepts of periodontal disease pathogenesis and confer diagnostic and prognostic value^2,3^. During progression of periodontitis both bacterial proteases i.e., gingipains from *Porphyromonas gingivalis *(*P. gingivalis*) and host-derived proteolytic enzymes (i.e., neutrophile serine proteases) are secreted into oral milieu^4–7^. Monitoring proteases and their inhibitors in saliva could be useful to serve as molecular indicator in monitoring periodontal inflammatory processes at the clinical setting^5,6,8,9^.

Serine protease inhibitors including secretory leukocyte protease inhibitor (SLPI), elastase-specific inhibitor and squamous cell carcinoma antigen protect tissues from excessive damage by proteases during inflammation^10^. SLPI is an 11.7 kDa cationic protein that inhibits mainly neutrophile elastase, cathepsin G and trypsin, which are involved in inflammatory tissue destruction^11–13^. Besides its anti-protease activity, SLPI has also anti-microbial, anti-inflammatory and immunomodulatory effects^11,12^. It is known to inhibit bacterial growth and viral replication in monocytic cells and also protects the tissues against inflammatory products by down-regulating the macrophage responses against bacterial lipopolysaccharide (LPS)^11,13^.

SLPI has capacity to promote scarless wound healing and maintains epithelial integrity of junctional epithelium^14–16^. Into et al*.*^17^ and Laugisch et al*.*^18^ reported that reduced SLPI levels were found in inflamed gingival tissue and gingival crevicular fluid (GCF) of chronic periodontitis patients infected with *P. gingivalis.* The authors^17–19^ suggested that the degradation of SLPI by *P. gingivalis* gingipains might be responsible for reduced SLPI levels, decreased host protective capacity and periodontal tissue destruction in chronic periodontitis patients.

To date, little is known about the SLPI levels in saliva of patients with different forms of periodontal disease^20,21^. Previously, Cox et al*.*^20^ demonstrated that SLPI is present in whole saliva of patients with severe chronic periodontitis and reported that the levels of SLPI in saliva were significantly lower than in GCF obtained from the same patients. Hence, this is the first time that the levels of SLPI have been determined in saliva samples from the different diagnostic groups of patients and periodontally healthy individuals. The specific objective of the present study was to compare the salivary levels of SLPI between groups of healthy individuals and those with gingivitis and generalized stage 3 grade C periodontitis.

## Results

Saliva samples were obtained from 24 patients with generalized stage 3 grade C periodontitis, 24 patients with gingivitis, and 24 periodontally healthy individuals. The demographic characteristics and whole-mouth clinical periodontal parameters of the participants are presented in Table 1. There was no statistical difference in age or gender among periodontitis, gingivitis and periodontally healthy groups (p > 0.05). Periodontitis group had significantly higher mean probing depth (PD) and clinical attachment loss (CAL) values than gingivitis and periodontally healthy controls (p < 0.05). This group had also significantly higher mean percentage of sites with bleeding on probing (BOP (%)), papillary bleeding index (PBI), and plaque index (PI) scores compared to gingivitis and healthy groups (p < 0.05). Gingivitis group had significantly higher mean PD, BOP (%), PBI, and PI scores than healthy controls (p < 0.05).

**Table 1:** Demographic variables and whole-mouth clinical periodontal parameters of study groups.

Groups	Periodontitis (n = 24)	Gingivitis (n = 24)	Periodontal health (n = 24)
**Demographic variables**			
Age (years; mean ± SD)	37.95 ± 3.44	36.50 ± 4.96	36.12 ± 4.73
Sex (n)			
Females	12	13	11
Males	12	11	13
**Periodontal parameters**			
PD (mm)	4.37 ± 0.46*	2.14 ± 0.27^**‡**^	1.67 ± 0.22
CAL (mm)	5.43 ± 0.69*	0.01 ± 0.01	0.01 ± 0.01
BOP (%)	81.56%*	67.34%^**‡**^	2.99%
PBI	2.72 ± 0.39*	2.22 ± 0.26^**‡**^	0.19 ± 0.15
PI	3.73 ± 0.27*	3.11 ± 0.41^**‡**^	1.81 ± 0.48

All data (except for sex) are given in terms of mean ± SD. *p < 0.05, significantly higher than the gingivitis and healthy groups. ^‡^p < 0.05, significantly higher than the healthy group.

*PD* probing depth, *CAL* clinical attachment loss, *BOP (%)* the percentage of the bleeding on probing, *PBI* papillary bleeding index, *PI* plaque index, *SD* standard deviation, *n* number, *mm* millimeter.

Salivary SLPI concentrations (ng/ml) from enzyme-linked immunosorbent assay (ELISA) readings of the studied groups are demonstrated in Table 2. SLPI concentration was detected in all saliva samples, ranging from 23.81 nanogram (ng)/milliliter (ml) to 315.56 ng/ml. Periodontitis group had lower salivary SLPI concentrations (ng/ml) compared to gingivitis group (4.04-fold) (p < 0.05) and similar salivary SLPI concentrations (ng/ml) to periodontally healthy group (p > 0.05). There was no significant difference between gingivitis and periodontally healthy group in terms of salivary SLPI concentrations (ng/ml) (p > 0.05).

**Table 2:** Salivary SLPI concentrations and total protein levels of the study groups.

Analytes	Periodontitis	Gingivitis	Periodontal health
SLPI levels (ng/ml)	35.66 (24.53–56.50)*	143.91 (24.90–315.56)	40.39 (23.81–308.77)
Normalized SLPI levels (ng/mg protein)	39.39 (26.59–63.39)^**†**^	190.75 (25.99–465.42)	72.01 (30.66–462.23)
Total protein (mg/ml)	0.83 (0.73–0.98)	0.78 (0.50–0.97)	0.65 (0.42–0.92)^**‡**^

Data are expressed as median (minimum–maximum). *Significantly different from gingivitis group (p < 0.05). ^†^Significantly different from gingivitis and periodontally healthy group (p < 0.05).

^‡^Significantly different from gingivitis and periodontitis group (p < 0.05).

*SLPI* secretory leukocyte protease inhibitor, *ng* nanogram, *mg* milligram, *ml* milliliter.

Salivary SLPI concentrations (ng/ml) from ELISA readings were normalized for total protein concentrations (milligram (mg)/ml) because salivary flow rate may reflect differences in composition. Total protein concentrations (mg/ml) were detected in all saliva samples, ranging from 0.42 to 0.98 mg/ml. Salivary total protein concentrations (mg/ml) and normalized salivary SLPI levels (ng/mg protein) in study groups are shown in Table 2. Salivary total protein concentrations of periodontitis and gingivitis groups were significantly higher than periodontally healthy group (p < 0.05). Periodontitis and gingivitis groups had similar total protein concentrations (p > 0.05). Normalized salivary SLPI levels were significantly lower in periodontitis group compared to gingivitis (4.84-fold) and healthy controls (1.83-fold) (p < 0.05). Gingivitis group had higher normalized salivary SLPI levels than healthy controls however the difference did not reach to statistical significance (p > 0.05).

**Figure 1:**
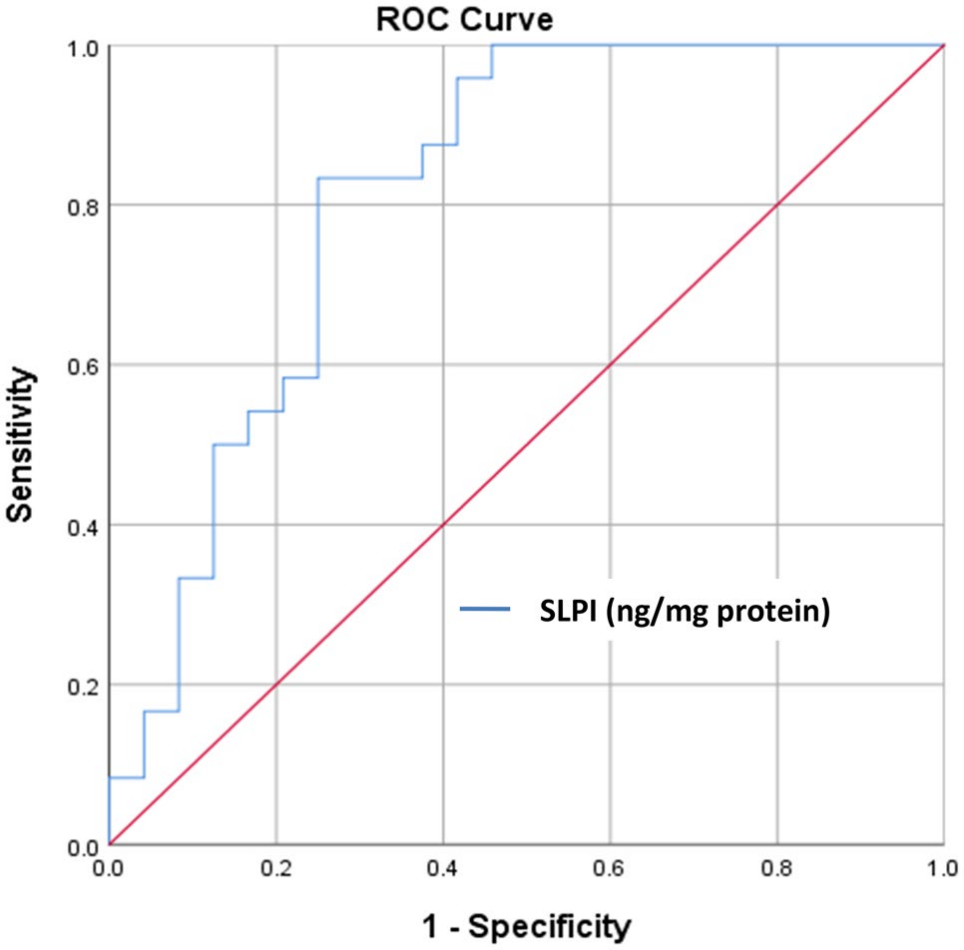
ROC curve for normalized salivary SLPI levels to identify periodontitis versus periodontal health. AUC: 0.81 with 83% sensitivity and 75% specificity (95% CI for the AUC: 0.68–0.93). *ROC* receiver operating characteristic, *SLPI* secretory leukocyte protease inhibitor, *CI* confidence interval, *AUC* area under the curve, *ng* nanogram, *mg* milligram.

**Figure 2:**
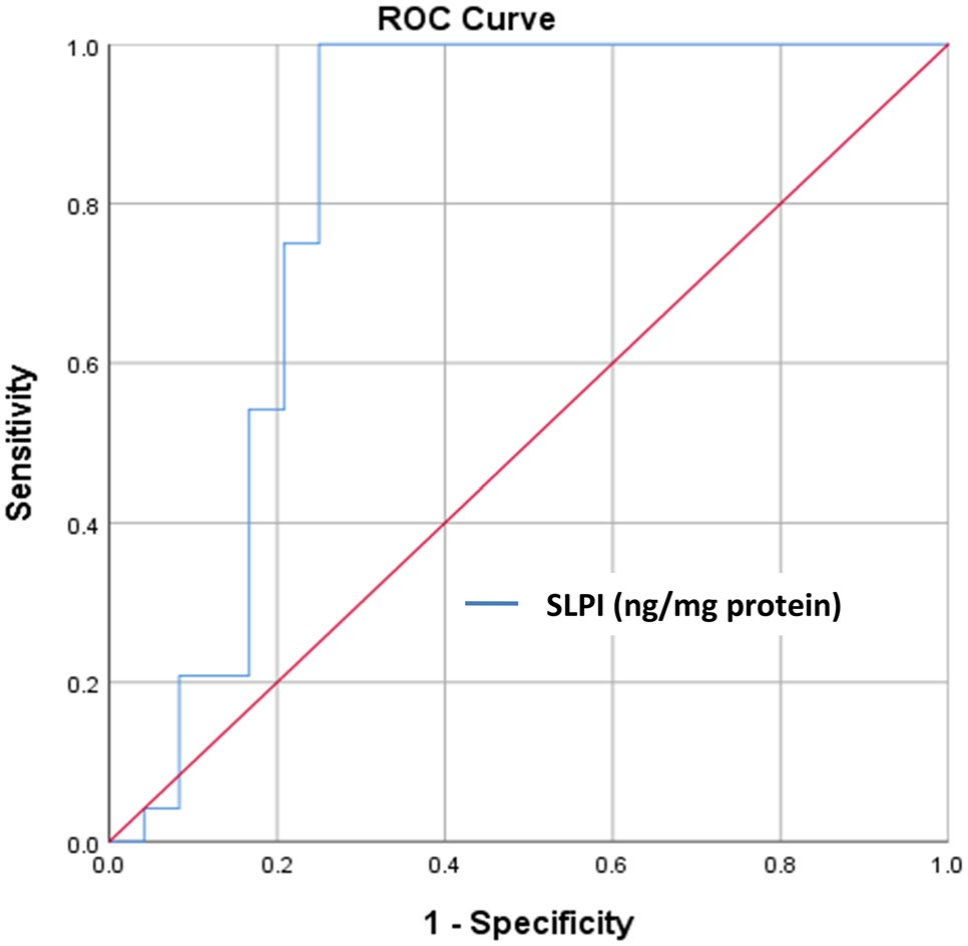
ROC curve for normalized salivary SLPI levels to identify periodontitis versus gingivitis. AUC: 0.82 with 100% sensitivity and 75% specificity (95% CI for the AUC: 0.68–0.96). *ROC* receiver operating characteristic, *SLPI* secretory leukocyte protease inhibitor, *CI* confidence interval, *AUC* area under the curve, *ng* nanogram, *mg* milligram.

Correlations of salivary SLPI levels with clinical periodontal parameters are shown in Table 3. Normalized salivary SLPI levels (ng/mg protein) were negatively correlated with all clinical periodontal parameters (p < 0.05 for all). Additionally, plotted receiver operating characteristics (ROC) curve showed good diagnostic value for normalized salivary SLPI levels (ng/mg protein) for diagnosis of periodontitis versus periodontal health (area under the curve (AUC): 0.81) (Table 4, Fig. 1). Selected cut-off value was 55 ng/mg protein for SLPI. Normalized salivary SLPI levels (ng/mg protein) showed 83.3% sensitivity and 75% specificity and 76.9% positive predictive value. Plotted ROC curve also showed good diagnostic value for normalized salivary SLPI levels (ng/mg protein) for diagnosis of periodontitis versus gingivitis (AUC: 0.82) (Table 5, Fig. 2). Selected cut-off value was 65 ng/mg protein for SLPI. Normalized salivary SLPI levels (ng/mg protein) showed 100% sensitivity and 75% specificity and 80% positive predictive value.

**Table 3:** Correlations of salivary SLPI levels with clinical periodontal parameters.

Variables	Age (years)	PD (mm)	CAL (mm)	BOP (%)	PBI	PI
**SLPI levels (ng/ml)**						
r	− 0.016	− 0.205	− 0.330*	− 0.243**†**	− 0.196	− 0.129
p	0.895	0.083	0.005	0.040	0.098	0.281
**Normalized SLPI levels (ng/mg protein)**						
r	0.032	− 0.356*	− 0.394*	− 0.404*	− 0.352*	− 0.285**†**
p	0.789	0.002	0.001	< 0.001	0.002	0.015

According to Spearman's rank correlation test, r: correlation coefficient and p: 2-tailed significance level. *Correlation is significant at 0.01 level. ^†^Correlation is significant at 0.05 level.

*PD* probing depth, *CAL* clinical attachment loss, *BOP (%)* the percentage of the bleeding on probing, *PBI* papillary bleeding index, *PI* plaque index, *SLPI* secretory leukocyte protease inhibitor, *ng* nanogram, *mg* milligram, *ml* milliliter, *mm* millimeter.

**Table 4:** AUC and cut-off value for normalized salivary SLPI levels to identify periodontitis (comparing periodontitis to periodontal health).

Analyte	AUC	% 95 CI	Cut-off (ng/mg protein)	p value	Sensitivity (%)	Specificity (%)	Positive predictive value (%)	Negative predictive value (%)
SLPI (ng/mg protein)	0.81	0.68–0.93	< 55	< 0.001	83.3	75.0	76.9	81.8

Positive and negative predictive values were given as percentage and p values are calculated by χ^2^ test.

*AUC* area under the curve, *CI* confidence interval, *SLPI* secretory leukocyte protease inhibitor, *ng* nanogram, *mg* milligram.

**Table 5:** AUC and cut-off value for normalized salivary SLPI levels to identify periodontitis (comparing periodontitis to gingivitis).

Analyte	AUC	% 95 CI	Cut-off (ng/mg protein)	p value	Sensitivity (%)	Specificity (%)	Positive predictive value (%)	Negative predictive value (%)
SLPI (ng/mg protein)	0.82	0.68–0.96	< 65	< 0.001	100.0	75.0	80.0	100.0

Positive and negative predictive values were given as percentage and p values are calculated by χ^2^ test.

*AUC* area under the curve, *CI* confidence interval, *SLPI* secretory leukocyte protease inhibitor, *ng* nanogram, *mg* milligram.

## Discussion

The present cross-sectional study is the first to investigate the involvement of SLPI in generalized stage 3 grade C periodontitis demonstrating that SLPI levels are reduced in periodontitis, compared with health or gingivitis. Accordingly, normalized SLPI concentrations in saliva were lower in patients with periodontitis than in those with gingivitis. Moreover, a negative correlation was demonstrated between SLPI levels and clinical periodontal parameters. These findings could suggest a potential association of SLPI with clinical presentation of periodontitis and its reduced concentrations observed in patients with severe periodontitis may constitute a biological mechanism that promotes progressive tissue loss.

To date, there is very limited data on the salivary SLPI levels in systemically healthy periodontitis patients and also about the diagnostic capacity of salivary levels of this inhibitor in periodontal diseases^20,21^. Therefore, in this study, whole saliva was used to evaluate SLPI levels of individuals with periodontitis. Salivary SLPI concentrations from ELISA readings were also normalized for total protein content because salivary flow rate may reflect differences in composition^5,22–24^. For that reason, the discussion was based mainly on the normalized salivary SLPI levels in the present study.

It has been known that SLPI is also expressed by secretory cells in the salivary glands^11–13^. Therefore, glandular saliva is expected to be a major source of SLPI in whole saliva. However, Cox et al*.*^20^ reported that the concentration of SLPI was actually higher in GCF than in whole saliva, and the level of SLPI in whole saliva was correlated with those in GCF rather than in parotid saliva. The increase in salivary SLPI levels during gingivitis may have been contributed from GCF^25^.

Periodontal tissue is continuously exposed to dental biofilm bacteria, hence a robust innate immune response is crucial to maintain periodontal homeostasis^26,27^. The innate immune response, which is the first line of defense, is able to recognize invading pathogens and trigger immune responses to eliminate them^27,28^. SLPI is an anti-microbial peptide and a member of the innate immunity-associated proteins^11,12^. It is constitutively expressed in gingival epithelial cells and junctional epithelium, with the level of expression stimulated by LPS, neutrophil elastase, and proinflammatory cytokines^14,15,29,30^. SLPI plays a critical role to maintain epithelial integrity in gingival tissues and periodontal tissue homeostasis^14,15^. Additionally, neutrophiles that constitute a major defense mechanism against extension of plaque into the gingival sulcus, also produce SLPI^27,29^. In the present study, the higher normalized salivary SLPI levels of periodontally healthy individuals than the periodontitis patients might be partially explained by the stimulation of SLPI as a part of natural immunity in periodontal tissues.

Minami^31^ demonstrated that GCF SLPI levels were significantly higher in sites with periodontal inflammation compared to healthy sites in patients with periodontitis. The author^31^ speculated that SLPI production by the pocket epithelium could potentially be stimulated by LPS from subgingival pathogenic bacteria, elastase from neutrophils and cytokines from the inflamed periodontal tissues. Contrary to Minami^31^, it has been suggested that reduced SLPI levels were found in GCF and gingival tissue of chronic periodontitis patients infected with *P. gingivalis*^17,18,32,33^. The authors^17,18,32–35^ speculated that arginine-specific gingipains from *P. gingivalis* degraded and inhibited the expression of SLPI. Kretschmar et al*.*^32^ reported that GCF SLPI levels were also reduced in periodontal patients without detectable level of *P. gingivalis.* SLPI and elastase levels have been reported to be inversely correlated in periopatients^20^. Increased levels of neutrophile proteases (elastase, protease 3 and cathepsin B) can contribute to significant depletion of SLPI in the infected periodontitis sites in those patients^18,32,36^. Pateel et al*.*^21^ speculated that lower salivary SLPI levels in chronic periodontitis may be explained by the degradation of SLPI by cathepsins and bacterial cysteine proteases. Therefore, in the present study, when compared to periodontal health and gingivitis, reduced normalized salivary SLPI levels in generalized stage 3 grade C periodontitis might be due to the proteolytic cleavage of this inhibitor by *P. gingivalis* gingipain and neutrophil proteases.

In the current study, there was a trend towards an increase in both salivary SLPI concentrations from ELISA readings and normalized salivary SLPI levels in patients with gingivitis when compared to periodontally healthy individuals. In gingivitis, concomitant with the increase in plaque biomass and gingival inflammation, SLPI could potentially be induced by bacterial LPS, neutrophil elastase and cytokines as a host protective mechanism^31^. On the other hand, in this study, periodontitis patients had significantly lower salivary SLPI concentrations from ELISA readings and normalized salivary SLPI levels than in those of patients with gingivitis. It has been generally accepted that there is microbial community diversity between gingivitis and periodontitis^37,38^. Gingivitis develops via the bacterial components of subgingival biofilm shift from Gram-positive streptococci to Gram-negative anaerobes such as *Actinobacillus, Fusobacterium, Prevotella, Capnocytophaga, Campylobacter* and *Eikenella*^37,38^. On the other hand, *P. gingivalis* is a major keystone pathogen involved in periodontitis capable of causing tissue damage, and can be detected in up to 78% of diseased individuals^39,40^. Kretschmar et al.^32^ found that GCF SLPI concentration decreased in periodontitis group and was negatively associated with *P. gingivalis* counts in dental plaque. Into et al.^17^ reported that the proteolytic activity of *P.* gingivalis gingipains (especially isoform RgpA) may be involved in the reduced level of protease inhibitors including SLPI in periodontitis patients. Yin et al*.*^35^ demonstrated that exposure to *Fusobacterium nucleatum *(*F. nucleatum*) induced the expression of protease inhibitors, while *P. gingivalis* degraded these protease inhibitors most effectively. During the formation of dental plaque, host-derived protease inhibitors may be stimulated by the presence of *F. nucleatum*, however may be rendered ineffective once protease-secreting bacteria are established^32^. As microbiological analysis was not performed in this study, no judgement can be made on this point. However, future mechanistic studies will reveal the relationships between oral microbial diversity and SLPI levels in periodontal disease at the molecular level.

SLPI plays a role as an “alarm” anti-protease mediating anti-inflammatory, immunomodulatory and anti-microbial effects^12^. It has been known that SLPI inhibits LPS-induced nuclear factor kappa B (NF-κB) signaling pathway via the modulation of Toll-like receptor-2 and -4 expression and downregulates the production of pro-inflammatory cytokines tumor necrosis factor-alpha, interleukin-6 and -8 and monocyte chemoattractant protein-1^41,42^. In periodontitis, the degradation of SLPI by *P. gingivalis* gingipains and neutrophil proteases may lead to the LPS induced-NF-κB activation and proinflammatory cytokine expression in gingival fibroblasts, which are closely related to periodontal breakdown^32,35,36,41,42^. In the current study, decreased salivary SLPI levels in patients with periodontitis might be associated with increased NF-κB pathway activation and proinflammatory cytokine expression through the cleavage of this inhibitor.

To the best of our knowledge, this is the first study to investigate the diagnostic potential of SLPI in periodontal diseases, although previous studies have examined other candidate biomarkers ^2,43–45^. Normalized salivary SLPI levels showed good diagnostic values to discriminate periodontitis from gingivitis (an AUC value of 0.82 with 100% sensitivity and 75% specificity) and periodontal health (an AUC value of 0.81 with 83% sensitivity and 75% specificity). These findings may suggest that salivary levels of SLPI could aid in identifying individuals with periodontitis. As the study could be limited by sample size, its cross-sectional nature and lack of microbiological data as well as inclusion of only systemically well controlled groups, further longitudinal studies in larger mixed cohorts are warranted.

## Conclusions

Within the limitations of this study, the present findings indicate that the levels of SLPI are high in periodontal health, further elevated in gingivitis, but eventually decreased in severe periodontitis beyond the former two states. This observation may have broader implications in the context of inflammatory diseases affecting the oral mucosa, as it shows that the bacterial burden is disturbing the homeostatic balances of anti-microbial and anti-protease factors in the oral cavity.

## Materials and methods

### Study population and clinical examination

A total of 72 individuals (36 females and 36 males; age range 26–43 years) seeking dental treatment in the School of Dentistry, Aydın Adnan Menderes University, Aydın, Turkey, were included for this cross-sectional study in 2015. The study design was approved by the Ethics Committee of the School of Medicine, İzmir Ege University with the protocol number (No: 19-12T/57) and the ethical principles of medical research outlined in Declaration of Helsinki, as revised in 2013 were followed. The purpose and procedures of the study were clarified to each participant and a signed informed consent was provided from every participant before starting the study.

According to the diagnostic criteria proposed by the 2017 International Workshop on the Classification of Periodontal and Peri-implant Diseases and Conditions^48^ participants were categorized into three distinct groups based upon their periodontal status: (1) 24 patients with generalized stage 3 grade C periodontitis; (2) 24 patients with gingivitis; and (3) 24 periodontally healthy individuals. Medical and dental histories were taken from all volunteers with an oral examination. All participants were never smokers and had at least 20 natural teeth. Patients were excluded from the study if they had any chronic inflammatory conditions such as diabetes mellitus, rheumatoid arthritis, cardiac, renal, hepatic and respiratory diseases and hematologic, immunologic or mucocutaneous disorders. Individuals who took medications such as immunosuppressive agents, antibiotics and anti-inflammatory drugs within the past 6 months were also excluded. None of the females were pregnant or lactating. Participants who had received any periodontal intervention within the preceding year and those who had removable partial dentures, orthodontic appliances or endodontic and restorative treatment requirements were not included in this study.

Probing depth (PD), clinical attachment loss (CAL), the percentage of sites with bleeding on probing (BOP %), papillary bleeding index (PBI)^46^, and plaque index (PI)^47^ were assessed during periodontal clinical examinations. Apart from PBI, all measurements were taken at six sites (mesio-buccal, mid-buccal, disto-buccal, mesio-palatal/lingual, mid-palatal/lingual, and disto-palatal/lingual) per tooth, except the third molars, using a manual periodontal probe (William’s periodontal probe, Hu-Friedy, Chicago, IL). PD and CAL were determined by measuring the distance from the base of the sulcus/pocket to the gingival margin and to the cement-enamel junction, respectively. The radiographic alveolar bone loss was evaluated on the digital panoramic radiograph in every participant. Periodontal status was assessed by two periodontists who were calibrated examiners (B.A. and V.Ö.Ö.). Before the study, inter-examiner calibration exercises for probing measurements (PD and CAL) were performed in five patients with a manual periodontal probe (William’s periodontal probe, Hu-Friedy, Chicago, IL). Every patient was examined by both examiners in two separate sessions one week apart. PD and CAL were measured on six surfaces of each tooth. Inter-examiner calibration was analyzed using κ-Cohen test and inter-examiner κ values were 0.88 (PD) and 0.85 (CAL).

Periodontitis patients had interproximal CAL ≥ 5 mm at 30% of the teeth or more. Care was taken to ensure that CAL was not caused by non‐periodontal causes such as gingival recession of traumatic origin, the presence of CAL on the distal aspect of a second molar due to extraction of a third molar, an endodontic lesion draining through the marginal periodontium and the occurrence of a vertical root fracture and dental caries extending in the cervical area of the tooth. These patients had also radiographic bone loss extending to the mid-third of the root or beyond and PD ≥ 6 mm at 30% of the teeth or more as well as Class II–III furcation involvement. These patients showed no more than four teeth loss due to periodontitis. The periodontitis grade of patients was estimated with indirect evidence of progression through % bone loss/age. Radiographic bone loss was determined from the tooth exhibiting the most extensive bone loss as a percentage of root length and % bone loss/age was calculated. If this value was > 1.0, participants were included in grade C. Gingivitis patients showed PD ≤ 3 mm with BOP ≥ 30% in the entire mouth as well as no interproximal CAL or alveolar bone loss. Periodontally healthy individuals in the control group had an intact periodontium (without detectable CAL or radiographic bone loss) or a reduced periodontium in a non-periodontitis patient (without interproximal CAL or radiographic bone loss). In this group, PD was ≤ 3 mm and BOP was < 10%.

### Saliva collection and processing

Saliva sampling was performed a day after the periodontal examination. Unstimulated whole saliva was obtained from the participants in the morning between 8:00 am and 10:00 am. A modification of the method described by Navazesh^49^ was used for saliva collection. All individuals were requested to refrain from any oral hygiene practices such as toothbrushing, interdental cleaning and rinsing with mouthwash, as well as eating and drinking, for 2 h before collection. Every participant was asked first to rinse the mouth completely with tap water for 2 min and to wait for 10 min. Then, the participants were requested to let the saliva pool in the floor of their mouth and to allow the saliva to drain passively into a sterile plastic container for 5 min. Saliva samples were kept on ice and then stored at – 40 °C, until further analysis.

### Measurement of SLPI levels in saliva samples

On the day of analysis, frozen saliva samples were thawed on ice and centrifuged at 10,000×*g* for 15 min at 4 °C. Obtained supernatants were supplemented with EDTA-free protease inhibitor cocktail (Roche Applied Science, Basel, Switzerland). Total protein levels were measured by colorimetric detection method (Pierce BCA Protein Assay Kit, Thermo Fisher Scientific, Basel, Switzerland). Briefly, 25 µl standard (bovine serum albumin) or supernatants were pipetted into a microplate well, and 200 µl working reagent was added. After 30 s of shaking, the plate was incubated at 37 °C for 30 min. Absorbance was read at 562 nm on a plate reader after cooling at room temperature. A four-parameter standard curve was used to determine total protein concentrations in saliva. Results are expressed as mg/ml. The SLPI levels in saliva samples were measured by ELISA (Human SLPI DuoSet ELISA, R&D Systems, Abingdon, UK) using commercial kits according to manufacturer’s guidelines. Collected supernatants were diluted 1000-fold before plating out. Minimum detection limits in assays were 15.6 pg/ml for SLPI. Absorbance was read at 562 nm on a plate reader and cytokine concentrations were determined from the standard curve. Salivary SLPI concentrations from ELISA readings were presented as ng/ml. Furthermore, salivary SLPI concentrations were normalized by total protein concentrations and expressed as ng/mg protein.

### Statistical analysis

The sample size was calculated using a specialized software package (G*Power version 3.0.8, Heinrich Heine University, Düsseldorf) for power analysis. Considering a statistical significance among three groups for salivary SLPI levels at 0.40 f-type effect size, 0.05 type I error, and 80% power using One-Way ANOVA method, the minimum sample size required was 22 per group.

All data analyses were performed using a statistical package IBM SPSS Statistic for Windows, Version 25.0 (Released 2017, Armonk, NY: IBM Corp.). Normality of the data was checked by Shapiro Wilk’s normality test. Comparisons of clinical parameters and salivary SLPI levels among the study groups were performed using Kruskal–Wallis test, and Dunn’s test (with Bonferroni correction) was used to pairwise comparisons for non-normally distributed variables. Receiver operating characteristic (ROC) curves were constructed to assess the ability of SLPI for diagnosis of periodontitis. Cut-off values were obtained using ROC curves. The area under the curve (AUC) was summary measure of accuracy of the analytes, with values between 0.7 and 0.8 indicative of acceptable discrimination, between 0.80 and 0.90 indicative of excellent discrimination, and more than 0.90 indicative of outstanding discrimination^50^. Chi-square analysis was used to compare the proportion of genders between groups. The correlations among salivary SLPI levels and clinical periodontal parameters were determined by Spearman rank correlation analysis. Statistical significance was considered at p < 0.05 for all the tests.

## Data Availability

The datasets generated and analyzed during this study are included in this published article or available from the corresponding author on reasonable request.
